# Supplementary material for: Selective deletion of Tsc1 from mouse cerebellar Purkinje neurons drives sex-specific behavioral impairments linked to autism
Source: Front Behav Neurosci. 2024 Dec 17;18:1474066. doi: 10.3389/fnbeh.2024.1474066 (PMC11685134; doi:10.3389/fnbeh.2024.1474066)
Supplement: Supplementary file 1 [file Data_Sheet_1.pdf]

Supplemental Table 1: Results of statistical analyses for 9-11 week-old mice.
Main effects of two-way ANOVA tests across sexes for each behavioral assay are reported as P-values. P<0.05 was considered to be statistically significant; significant effects are bolded. Dash indicates that a pairwise comparison was unable to be completed; RM, repeated measures.

N	Test	Test Details	*Post hoc* Multiple Comparisons
		**Elevated Balance Beam**	
N=21<br>Males=11<br>Females = 10	CN Males & Females<br>Time to Cross (10mm):<br>RM 2-way ANOVA	day x sex: F(3,57)=0.8883, P=0.4528<br>sex: F(1,19)=0.3849, P=0.5424<br>day: F(2.592,49.24)=0.8088, P=0.4794	Šídák's, Male vs. Female<br>Day 1: P=0.5957<br>Day 2: P=0.9999<br>Day 3: P=0.9954<br>Day 4: P=0.8159
N=20<br>Males=10<br>Females = 10	*Tsc1*$^{mut/wt}$ Males & Females<br>Time to Cross (10mm):<br>RM 2-way ANOVA	sex x day: F(3,54)=0.1186, P=0.9488<br>sex: F(1,18)=1.784, P=0.1983<br>day: F(2.078,37.41)=1.630, P=0.2089	Šídák's, Male vs. Female<br>Day 1: P=0.8600<br>Day 2: P=0.9072<br>Day 3: P=0.5291<br>Day 4: P=0.7677
N=24<br>Males=13<br>Females = 11	*Tsc1*$^{mut/mut}$ Males & Females<br>Time to Cross (10mm):<br>RM 2-way ANOVA	sex x day: F(3,66)=1.882, P=0.1412<br>sex: F(1,22)=2.828, P=0.1068<br>day: F(2.175,47.86)=1.630, P=0.2089	Šídák's, Male vs. Female<br>Day 1: P=0.1941<br>Day 2: P=0.1873<br>Day 3: P=0.8345<br>Day 4: P=0.9753
N=21<br>Males=11<br>Females = 10	CN Males & Females<br>Time to Cross (8mm):<br>RM 2-way ANOVA	sex x day: F(3,57)=0.08166, P=0.9697<br>sex: F(1,19)=4.365, P=0.0504<br>day: F(2.530,48.07)=0.1666, P=0.8912	Šídák's, Male vs. Female<br>Day 1: P=0.6696<br>Day 2: P=0.1831<br>Day 3: P=0.7248<br>Day 4: P=0.6880
N=20<br>Males=10<br>Females = 10	*Tsc1*$^{mut/wt}$ Males & Females<br>Time to Cross (8mm):<br>RM 2-way ANOVA	sex x day: F(3,54)=0.028667, P=0.9940<br>sex: F(1,18)=1.016, P=0.3269<br>day: F(2.805,50.49)=1.372, P=0.2627	Šídák's, Male vs. Female<br>Day 1: P=0.4348<br>Day 2: P=0.9786<br>Day 3: P=0.7831<br>Day 4: P=0.9490
N=24<br>Males=13<br>Females = 11	*Tsc1*$^{mut/mut}$ Males & Females<br>Time to Cross (8mm):<br>RM 2-way ANOVA	sex x day: F(3,66)=1.073, P=0.3667<br>sex: F(1,22)=0.04588, P=0.8324<br>day: F(1.938,42.63)=1.330, P=0.2747	Šídák's, Male vs. Female<br>Day 1: P=0.9623<br>Day 2: P=0.9119<br>Day 3: P=0.9853<br>Day 4: P=0.9845
N=21<br>Males=11<br>Females = 10	CN Males & Females<br>Foot-slips (10mm):<br>RM 2-way ANOVA	sex x day: F(3,57)=2.184, P=0.0986<br>sex: F(1,19)=0.3501, P=0.5610<br>day: F(2.029,38.55)=0.9767, P=0.3867	Šídák's, Male vs. Female<br>Day 1: P=0.8113<br>Day 2: P>0.9999<br>Day 3: P=0.5205<br>Day 4: --

N=20<br>Males=10<br>Females = 10	*Tsc1*$^{mut/wt}$ Males & Females<br>Foot-slips (10mm):<br>RM 2-way ANOVA	sex x day: F(3,54)=0.8319, P=0.4822<br>sex: F(1,18)=1.528, P=0.2323<br>day: F(2.201,39.62)=0.8319, P=0.4527	Šídák's, Male vs. Female<br>Day 1: P>0.9999<br>Day 2: P=0.5205<br>Day 3: --<br>Day 4: P=0.8142
N=24<br>Males=13<br>Females = 11	*Tsc1*$^{mut/mut}$ Males & Females<br>Foot-slips (10mm):<br>RM 2-way ANOVA	sex x day: F(3,66)=0.3565, P=0.7846<br>sex: F(1,22)=0.1423, P=0.7056<br>day: F(2.591,57)=2.834, P=0.0536	Šídák's, Male vs. Female<br>Day 1: P>0.9999<br>Day 2: P=0.9607<br>Day 3: P=0.9998<br>Day 4: P=0.9061
N=21<br>Males=11<br>Females = 10	CN Males & Females<br>Foot-slips (8mm):<br>RM 2-way ANOVA	sex x day: F(3,57)=1.182, P=0.3248<br>sex: F(1,19)=0.3073, P=0.5858<br>day: F(2.557,48.59)=0.9318, P=0.4204	Šídák's, Male vs. Female<br>Day 1: P=0.5953<br>Day 2: P=0.7766<br>Day 3: P=0.9931<br>Day 4: P=0.6969
N=20<br>Males=10<br>Females = 10	*Tsc1*$^{mut/wt}$ Males & Females<br>Foot-slips (8mm):<br>RM 2-way ANOVA	sex x day: F(3,54)=0.9649, P=0.4160<br>sex: F(1,18)=1.477, P=0.2400<br>day: F(2.6,46.8)=1.076, P=0.3621	Šídák's, Male vs. Female<br>Day 1: P=0.9981<br>Day 2: P=0.9960<br>Day 3: P=0.1967<br>Day 4: P=0.9090
N=24<br>Males=13<br>Females = 11	*Tsc1*$^{mut/mut}$ Males & Females<br>Foot-slips (8mm):<br>RM 2-way ANOVA	sex x day: F(3,66)=1.160, P=0.3318<br>sex: F(1,22)=0.02369, P=0.8791<br>day: F(2.539,55.86)=5.157, **P=0.0051**	Šídák's, Male vs. Female<br>Day 1: P=0.9985<br>Day 2: P=0.8292<br>Day 3: P=0.9969<br>Day 4: P=0.8586
		**Social Interaction**	
N=21<br>Males=11<br>Females = 10	CN Males & Females Direct Contacts (Approach):<br>RM 2-way ANOVA	sex x preference: F(1,19)=0.08960, P=0.7679<br>sex: F(1,19)=0.04783, P=0.8292<br>preference: F(1,19)=23.86, **P=0.0001**	Šídák's, Empty cup vs. unfamiliar animal<br>Male: **P=0.0071**<br>Female: **P=0.0040**
N=20<br>Males=10<br>Females = 10	*Tsc1*$^{mut/wt}$ Males & Females<br>Direct Contacts (Approach):<br>RM 2-way ANOVA	sex x preference: F(1,18)=1.994, P=0.1750<br>sex: F(1,18)=0.1828, P=0.6741<br>preference: F(1,18)=16.01, **P=0.0008**	Šídák's, Empty cup vs. unfamiliar animal<br>Male: P=0.1605<br>Female: **P=0.0025**
N=24<br>Males=13<br>Females = 11	*Tsc1*$^{mut/mut}$ Males & Females<br>Direct Contacts (Approach):<br>RM 2-way ANOVA	sex x preference: F(1,22)=1.639, P=0.6895<br>sex: F(1,22)=1.694, P=0.2065<br>preference: F(1,22)=19.11, **P=0.002**	Šídák's, Empty cup vs. unfamiliar animal<br>Male: P=0.1627<br>Female: P=0.0845

N=21<br>Males=11<br>Females = 10	CN Males & Females Direct Contacts (Novelty):<br>RM 2-way ANOVA	sex x preference: F(1,19)=3.604, P=0.0729<br>sex: F(1,19)=0.009826, P=0.9222<br>preference: F(1,19)=32.77, **P<0.0001**	Šídák's, Familiar animal vs. unfamiliar animal<br>Male: **P<0.0001**<br>Female: **P=0.0318**
N=20<br>Males=10<br>Females = 10	*Tsc1*$^{mut/wt}$ Males & Females Direct Contacts (Novelty):<br>RM 2-way ANOVA	sex x preference: F(1,18)=17.61, **P=0.0005**<br>sex: F(1,18)=4.992, **P=0.0384**<br>preference: F(1,18)=70.45, **P<0.0001**	Šídák's, Familiar animal vs. unfamiliar animal<br>Male: **P<0.0001**<br>Female: **P=0.0164**
N=24<br>Males=13<br>Females = 11	*Tsc1*$^{mut/mut}$ Males & Females Direct Contacts (Novelty):<br>RM 2-way ANOVA	sex x preference: F(1,22)=0.6437, P=0.4310<br>sex: F(1,22)=5.774, **P=0.0251**<br>preference: F(1,22)=30.97, **P<0.0001**	Šídák's, Familiar animal vs. unfamiliar animal<br>Males: **P=0.0002**<br>Females: **P=0.0076**
N=65<br>CN=11 Males, 10 Females<br>*Tsc1*$^{mut/wt}$ = 10 Males, 10 Females<br>*Tsc1*$^{mut/muut}$ = 13 Males, 11 Females	CN, *Tsc1*$^{mut/wt}$, & *Tsc1*$^{mut/mut}$, Males & Females<br>Percentage of Investigation (Approach): 2-way ANOVA	sex x genotype: F(2,59)=0.3772, P=0.6874<br>genotype: F(2,59)=3.220, **P=0.0471**<br>sex: F(1,59)=1.250, P=0.2681	Šídák's, Genotype<br>CN vs. *Tsc1*$^{mut/wt}$: P=0.6523<br>CN vs. *Tsc1*$^{mut/mut}$: **P=0.0424**<br>*Tsc1*$^{mut/wt}$ vs. *Tsc1*$^{mut/mut}$: P=0.4173
N=65<br>CN=11 Males, 10 Females<br>*Tsc1*$^{mut/wt}$ = 10 Males, 10 Females<br>*Tsc1*$^{mut/muut}$ = 13 Males, 11 Females	CN, *Tsc1*$^{mut/wt}$, & *Tsc1*$^{mut/mut}$, Males & Females<br>Percentage of Investigation (Novelty): 2-way ANOVA	sex x genotype: F(2,59)=2.922, P=0.0617<br>genotype: F(2,59)=1.239, P=0.2972<br>sex: F(1,59)=12.18, **P=0.0009**	Šídák's, Male vs. Female<br>CN: **P=0.0055**<br>*Tsc1*$^{mut/wt}$: **P=0.0434**<br>*Tsc1*$^{mut/mut}$: P=0.9986
**Repetitive Behavior**			
N=43<br>CN=6 Males, 8 Females<br>*Tsc1*$^{mut/wt}$ = 6 Males, 5 Females<br>*Tsc1*$^{mut/muut}$ = 9 Males, 9 Females	CN, *Tsc1*$^{mut/wt}$, & *Tsc1*$^{mut/mut}$, Males & Females<br>Total Time Grooming:<br>2-way ANOVA	sex x genotype: F(2,37)=2.414, P=0.1034<br>genotype: F(2,37)=0.2444, P=0.7844<br>sex: F(1,37)=4.682, **P=0.0370**	Šídák's, Male vs. Female<br>CN: P=0.0500<br>*Tsc1*$^{mut/wt}$: P=0.4180<br>*Tsc1*$^{mut/mut}$: P=0.9752

Supplemental Table 2: Results of statistical analyses for 16-24 week-old mice.

Main effects of two-way ANOVA tests across sexes for each behavioral assay are reported as P-values. P <0.05 was considered to be statistically significant; significant effects are bolded. Dash indicates that a pairwise comparison was unable to be completed; RM, repeated measures.

N	Test	Test Details	*Post hoc* Multiple Comparisons
		Elevated Balance Beam	
N=18<br>Males=10<br>Females=8	CN Males & Females Time to Cross (10mm):<br>RM 2-way ANOVA	sex x day: F(3,48)=0.6286, P=0.6001<br>sex: F(1,16)=1.279, P=0.2930<br>day: F(2.703,43.25)=0.2806, P=0.6036	Šídák's, Male vs. Female<br>Day 1: P=0.7256<br>Day 2: P= 0.9976<br>Day 3: P=0.9871<br>Day 4: P=0.9992
N=19<br>Males=9<br>Males=10	*Tsc1*$^{mut/mut}$ Males & Females Time to Cross (10mm):<br>RM 2-way ANOVA	sex x day: F(3,51)=0.7325, P=0.5374<br>sex: F(1,17)=2.595, P=0.12556<br>day: F(2.896,49.24)=1.746, P=0.1714	Šídák's, Male vs. Female<br>Day 1: P=0.3835<br>Day 2: P=0.9159<br>Day 3: P=0.4280<br>Day 4: P=0.6606
N=18<br>Males=10<br>Females=8	CN Males & Females Time to Cross (8mm):<br>RM 2-way ANOVA	sex x day: F(3,48)=0.77699, P=0.5126<br>sex: F(1,16)=0.2006, P=0.6602<br>day: F(2.719,43.5)=0.9122, P=0.4350	Šídák's, Male vs. Female<br>Day 1: P=0.9998<br>Day 2: P>0.9999<br>Day 3: P=0.6919<br>Day 4: P=0.9515
N=19<br>Males=9<br>Males=10	*Tsc1*$^{mut/mut}$ Males & Females Time to Cross (8mm):<br>RM 2-way ANOVA	sex x day: F(3,51)=0.1847, P=0.9063<br>sex: F(1,17)=1.010, P=0.3290<br>day: F(2.012,34.21)=2.271, P=0.1183	Šídák's, Male vs. Female<br>Day 1: P=0.9525<br>Day 2: P=0.8574<br>Day 3: P=0.6724<br>Day 4: P=0.8931
N=18<br>Males=10<br>Females=8	CN Males & Females Foot-slips (10mm):<br>RM 2-way ANOVA	sex x day: F(3,48)=1.183, P=0.3260<br>sex: F(1,16)=0.1616, P=0.6930<br>day: F(2.395,38.31)=0.3188, P=0.7668	Šídák's, Male vs. Female<br>Day 1: P=0.8222<br>Day 2: P=0.8142<br>Day 3: P=0.8142<br>Day 4: --
N=19<br>Males=9<br>Males=10	*Tsc1*$^{mut/mut}$ Males & Females Foot-slips (10mm):<br>RM 2-way ANOVA	sex x day: F(3,51)=1.904, P=0.1406<br>sex: F(1,17)=0.04234, P=0.8394<br>day: F(2.526,42.94)=1.1055, P=0.3510	Šídák's, Male vs. Female<br>Day 1: P=0.9978<br>Day 2: P=0.9772<br>Day 3: P=0.8486<br>Day 4: P=0.8643
N=18<br>Males=10<br>Females=8	CN Males & Females Foot-slips (8mm):<br>RM 2-way ANOVA	sex x day: F(3,48)=0.8063, P=0.4966<br>sex: F(1,16)=2.538, P=0.1307<br>day: F(2.618,41.89)=1.936, P=0.1454	Šídák's, Male vs. Female<br>Day 1: P=0.5121<br>Day 2: P=0.9507<br>Day 3: P=0.9081<br>Day 4: P=0.9998

N=19<br>Males=9<br>Males=10	*Tsc1*$^{mut/mut}$ Males & Females Foot-slips (8mm):<br>RM 2-way ANOVA	sex x day: F(3,51)=0.9218, P=0.4370<br>sex: F(1,17)=0.08584, P=0.7731<br>day: F(2.193,37.29)=1.280, P=0.2918	Šídák's, Male vs. Female<br>Day 1: P=0.9249<br>Day 2: P=0.9642<br>Day 3: P=0.9911<br>Day 4: P=0.9995
	Social Interaction		
N=14<br>Males=8<br>Females=6	CN Males & Females Direct Contacts (Approach):<br>RM 2-way ANOVA	sex x preference: F(1,12)=1.323, P=0.2725<br>sex: F(1,12)=2.937, P=0.1123<br>preference: F(1,12)=35.16, **P<0.0001**	Šídák's, Empty cup vs. unfamiliar animal<br>Male: **P=0.0003**<br>Female: **P=0.0163**
N=18<br>Males=9<br>Males=9	*Tsc1*$^{mut/mut}$ Males & Females Direct Contacts (Approach):<br>RM 2-way ANOVA	sex x preference: F(1,16)=8.096, **P=0.0117**<br>sex: F(1,16)=1.729, P=0.2071<br>preference: F(1,16)=58.68, **P<0.0001**	Šídák's, Empty cup vs. unfamiliar animal<br>Male: P=0.5288<br>Female: **P=0.0025**
N=14<br>Males=8<br>Females=6	CN Males & Females Direct Contacts (Novelty):<br>RM 2-way ANOVA	sex x preference: F(1,12)=0.2264, P=0.6427<br>sex: F(1,12)=3.252, P=0.0965<br>preference: F(1,12)=41.70, **P<0.0001**	Šídák's, Empty cup vs. unfamiliar animal<br>Male: **P=0.0004**<br>Female: **P=0.0038**
N=18<br>Males=9<br>Males=10	*Tsc1*$^{mut/mut}$ Males & Females Direct Contacts (Novelty):<br>RM 2-way ANOVA	sex x preference: F(1,16)=8.096, **P=0.0117**<br>sex: F(1,16)=1.729, P=0.2071<br>preference: F(1,16)=7.556, **P=0.0143**	Šídák's, Empty cup vs. unfamiliar animal<br>Male: P=0.1889<br>Female: P=0.0942
N=32<br>CN=8 Males, 6 Females<br>*Tsc1*$^{mut/mut}$=9 Males, 9 Females	CN & *Tsc1*$^{mut/mut}$ Males & Females Percentage of Investigation (Approach): 2-way ANOVA	sex x genotype: F(1,27)=5.713, **P=0.0241**<br>genotype: F(1,27)=6.065, **P=0.0205**<br>sex: F(1,27)=5.848, **P=0.0226**	Šídák's, Male vs. Female<br>CN: P=0.9998<br>*Tsc1*$^{mut/mut}$: **P=0.0026**
N=32<br>CN=8 Males, 6 Females<br>*Tsc1*$^{mut/mut}$=9 Males, 9 Females	CN & *Tsc1*$^{mut/mut}$ Males & Females Percentage of Investigation (Novelty): 2-way ANOVA	sex x genotype: F(1,27)=0.4497, P=0.5082<br>genotype: F(1,27)=12.92, **P=0.0013**<br>sex: F(1,27)=4.472, **P=0.0438**	Šídák's, Male vs. Female<br>CN: P=0.5644<br>*Tsc1*$^{mut/mut}$: P=0.0917

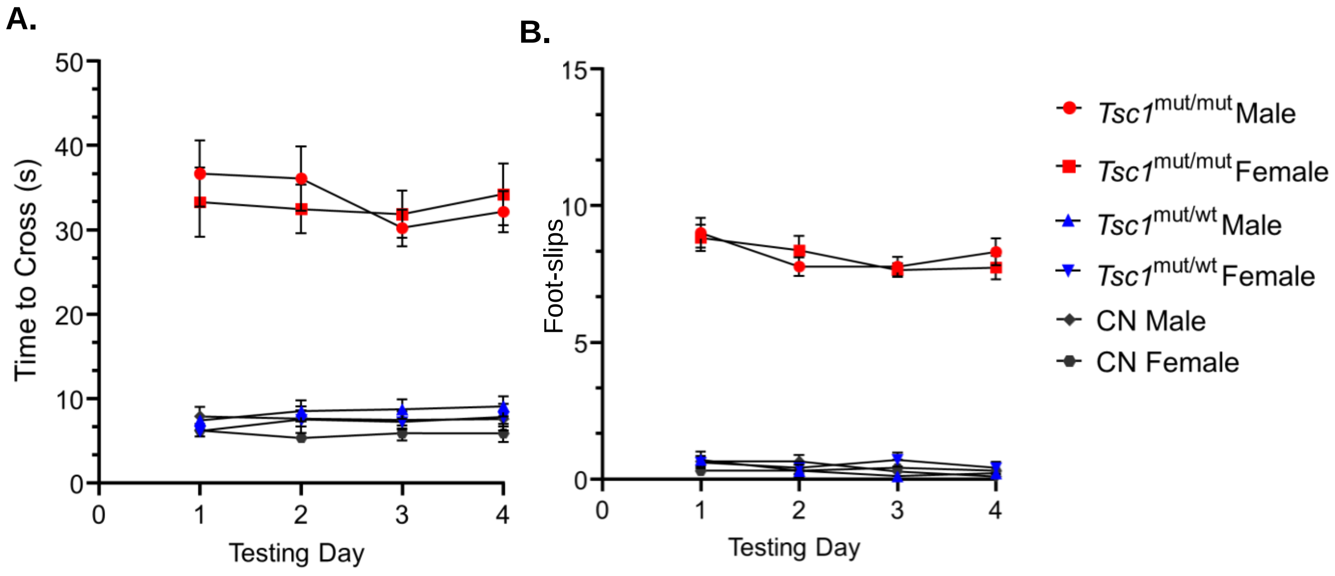

Supplemental Figure 1:
Motor coordination and balance testing of male and female 9–11 week-old *Tsc1*$^{mut/mut}$ (N=13 males and N=11 females), *Tsc1*$^{mut/wt}$ (N=10 males and N=10 females), and control (CN, N=11 males and N=10 females) mice were tested on an elevated balance beam 8mm in width. *Tsc1*$^{mut/mut}$ mice performed significantly ($p<0.0001$) worse than control animals on across all four testing days, but no difference in the time to cross (panel **A**) or number of hindlimb foot-slips (panel **B**) between sexes within each of the genotypes. Mean ± SEM values are plotted.

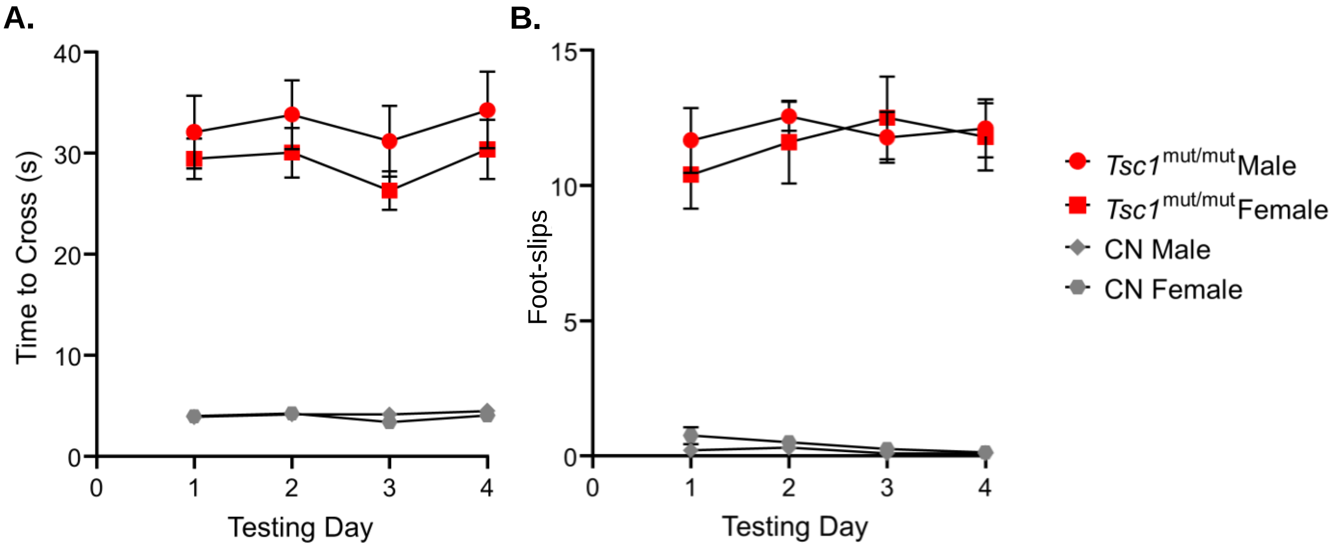

Supplemental Figure 2.
16-24 week-old *Tsc1*$^{mut/mut}$ (N=9 males and N=10 females) and control (N=10 males and N=8 females) mice of both sexes were tested on an elevated balance beam 8mm in width. *Tsc1*$^{mut/mut}$ mice performed significantly ($p<0.0001$) worse than control animals on across all four testing days, and no difference in the time to cross (panel **A**) or number of hindlimb foot-slips (panel **B**) between sexes was measured for either genotype. Mean ± SEM values are plotted.

A.

Male (postnatal day 16)

Tsc1$^{flox/flox}$; Cre (+)

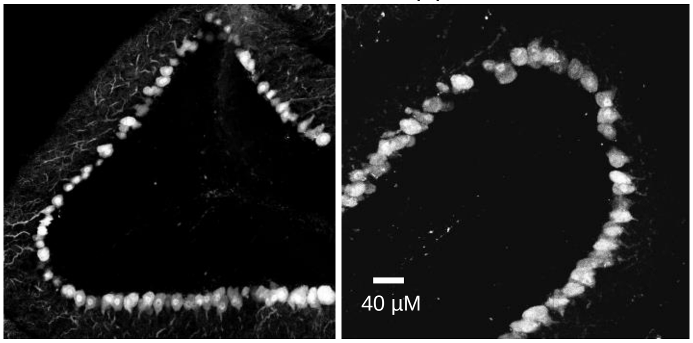

B.

Female (postnatal day 16)

Tsc1$^{flox/flox}$; Cre (+)

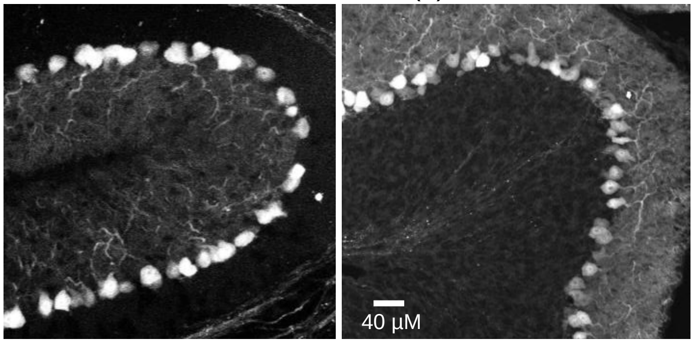

Supplemental Figure 3.
Using the Ai14 tdTomato Cre-reporter line (see Methods) tdTomato immunofluorescence was similarly expressed in the Purkinje neuron layer of parasagittal cerebellar sections taken from P16 male (panel **A.**) and P16 female (panel **B.**) *Tsc1*$^{flox/flox}$; Cre (+) animals.

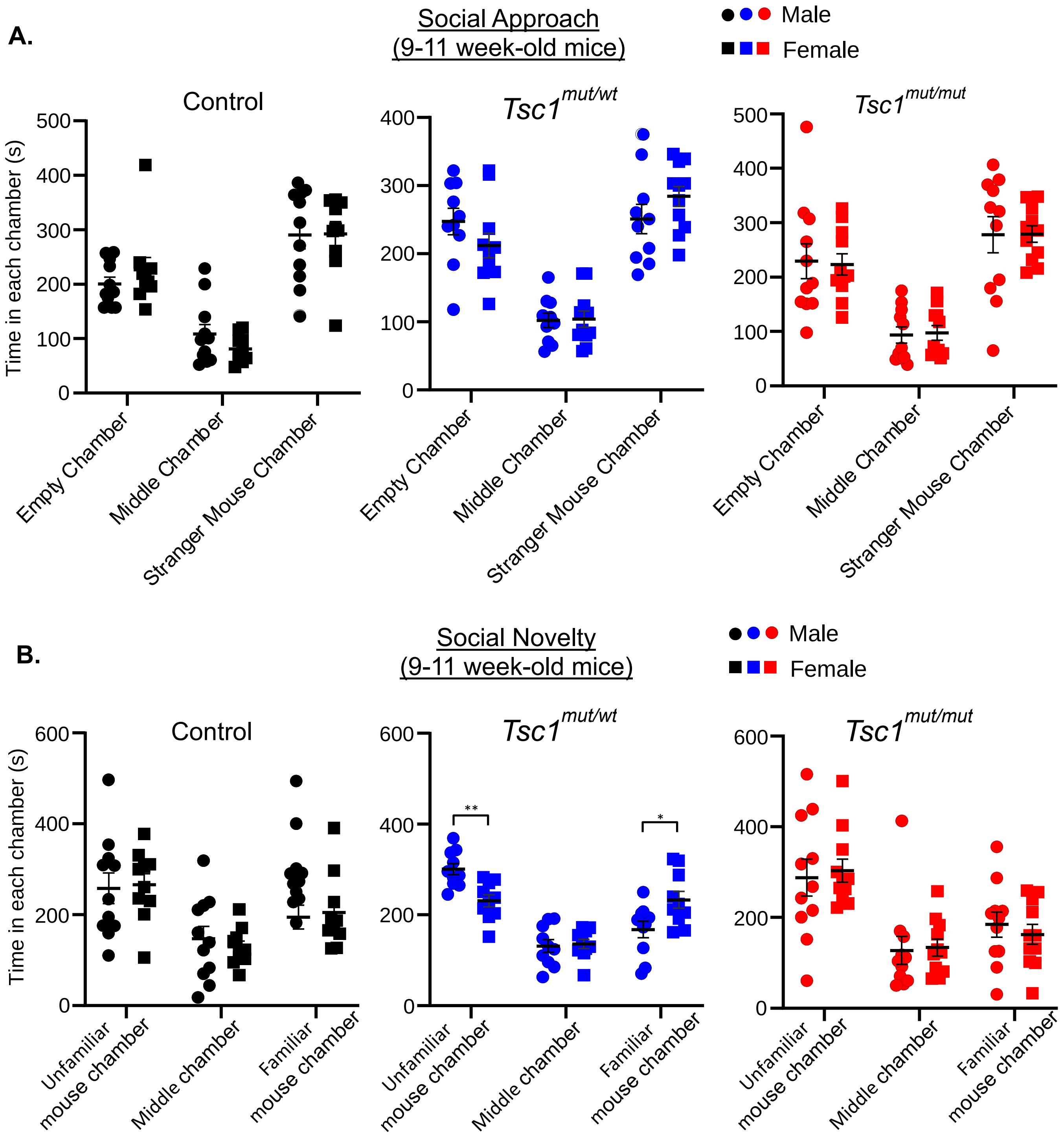

Supplemental Figure 4.

9-11 week-old control (*black,* left), *Tsc1*$^{mut/wt}$ (*blue*, center), and *Tsc1*$^{mut/mut}$ (*red*, right) mice of both sexes performed the 3-chamber social approach (**A.**) and social novelty (**B.**) assays for 10 minutes (presented in Figures 4 and 6). The time male and female mice spent in each chamber are plotted for each type of assay and across the three genotypes. Mean ± SEM values are plotted as black bars. Within each genotype for the social approach assay, there were no significant differences across male and female animals for the time spent in each chamber (Student's unpaired t-test). In the Social Novelty assay (panel **B.**), male *Tsc1*$^{mut/wt}$ animals spent significantly (P<.01) more time in the unfamiliar mouse chamber, and significantly (P<.05) less time in the familiar mouse chamber, when compared to female *Tsc1*$^{mut/wt}$ mice (unpaired Student's t-test). Using 3-way ANOVA analyses of time in chamber data (using genotype, chamber, and sex as major factors), we found chamber to be the only significant (P< .0001) major factor for each assay. No significant interactions between factors were measured in the social approach (**A.**) and social novelty assays (**B).**

Social Approach (16-24 week-old mice)

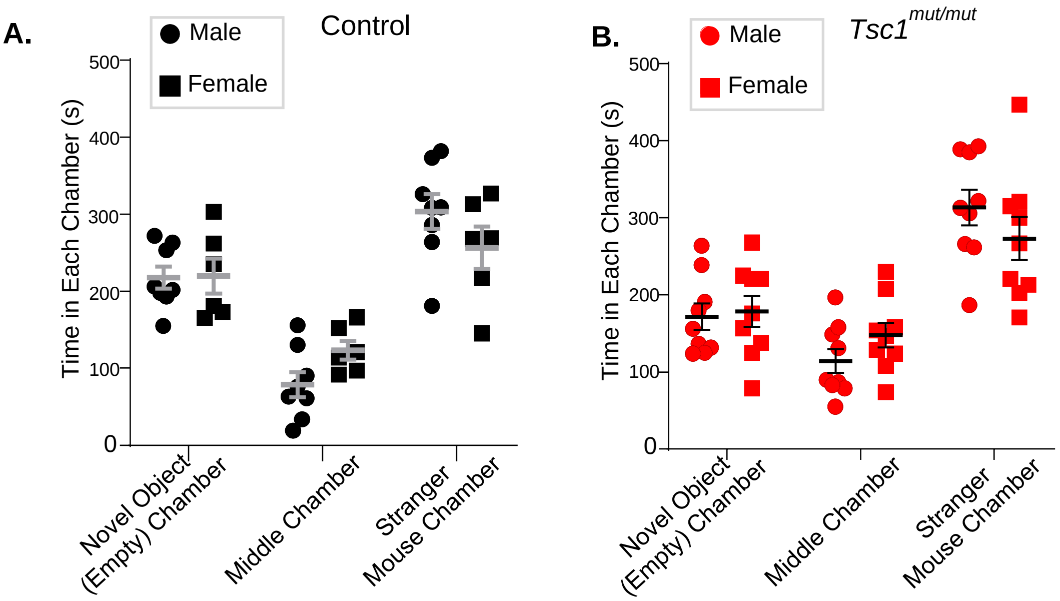

Social Novelty (16-24 week-old mice)

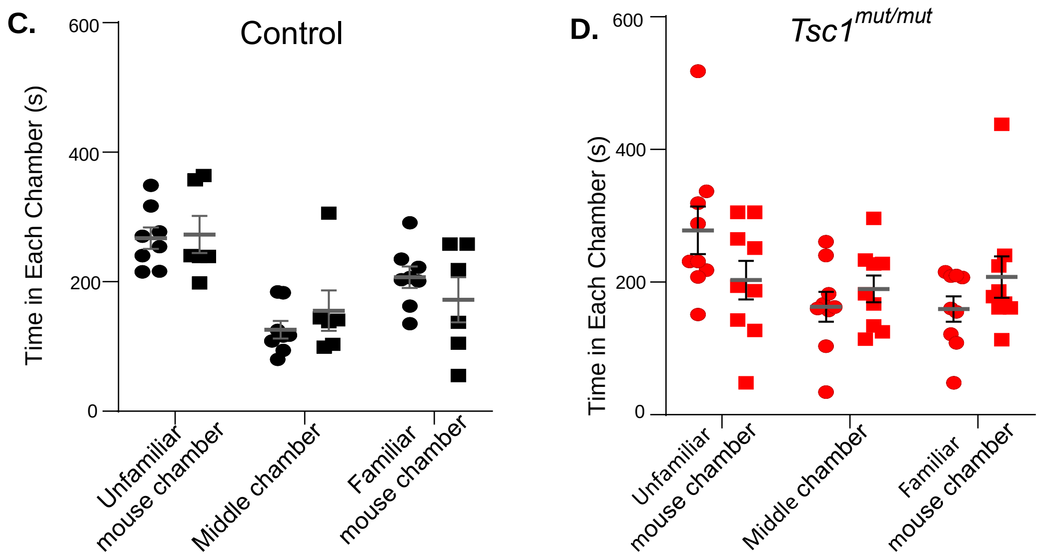

Supplemental Figure 5.
16-24 week-old control (*black*) and *Tsc1*$^{mut/mut}$ (*red*) mice performed the 3-chamber social approach and social novelty assays (presented in Figures 5 and 6, respectively). The time spent in each chamber during the 10-minute social approach assay (**A. B.**) and the 10-minute social novelty assay (**C. D.**) are plotted for each genotype. Mean ± SEM values are plotted as grey bars. Within each genotype, there were no significant differences between male and female animals for the time spent in each chamber during social approach or social novelty testing (Student's unpaired t-test). A 3-way ANOVA was used to assess these data across genotypes, using genotype, chamber, and sex as major factors. For social approach, this analysis revealed Chamber to be the only significant (P< .0001) major factor. A significant (P = .023) interaction was also identified between Chamber and Genotype factors. For social novelty (**C. D.**), three-way ANOVA analysis revealed Chamber to be the only significant (P< .0001) major factor with no significant interactions.
